# Supplementary figures and images for: Gliogenesis from the subventricular zone modulates the extracellular matrix at the glial scar after brain ischemia
Source: eLife. 2025 Aug 19;13:RP96076. doi: 10.7554/eLife.96076 (PMC12364484; doi:10.7554/eLife.96076)

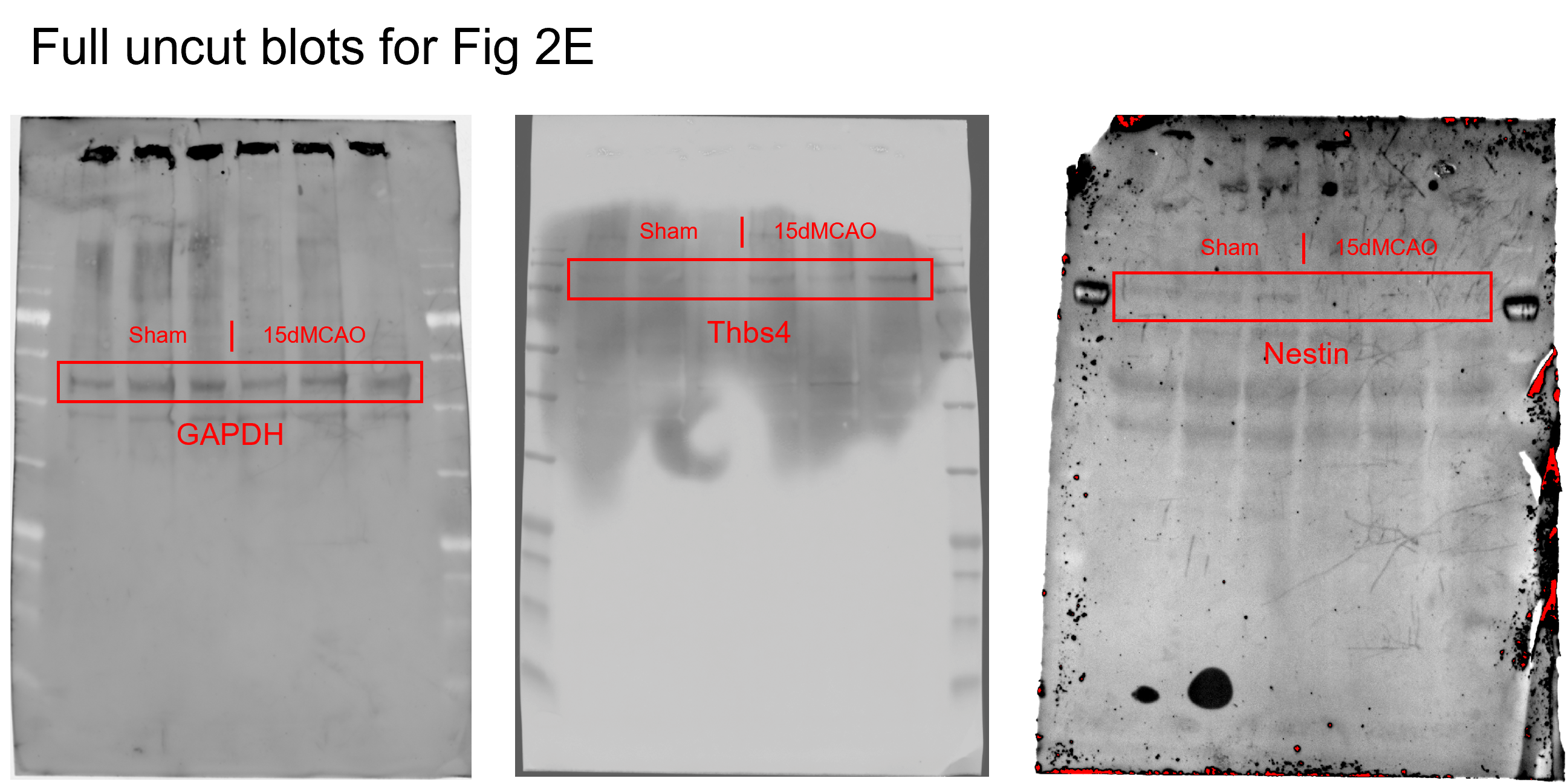

Supplement: Figure 2—source data 1. [file elife-96076-fig2-data1.tif]

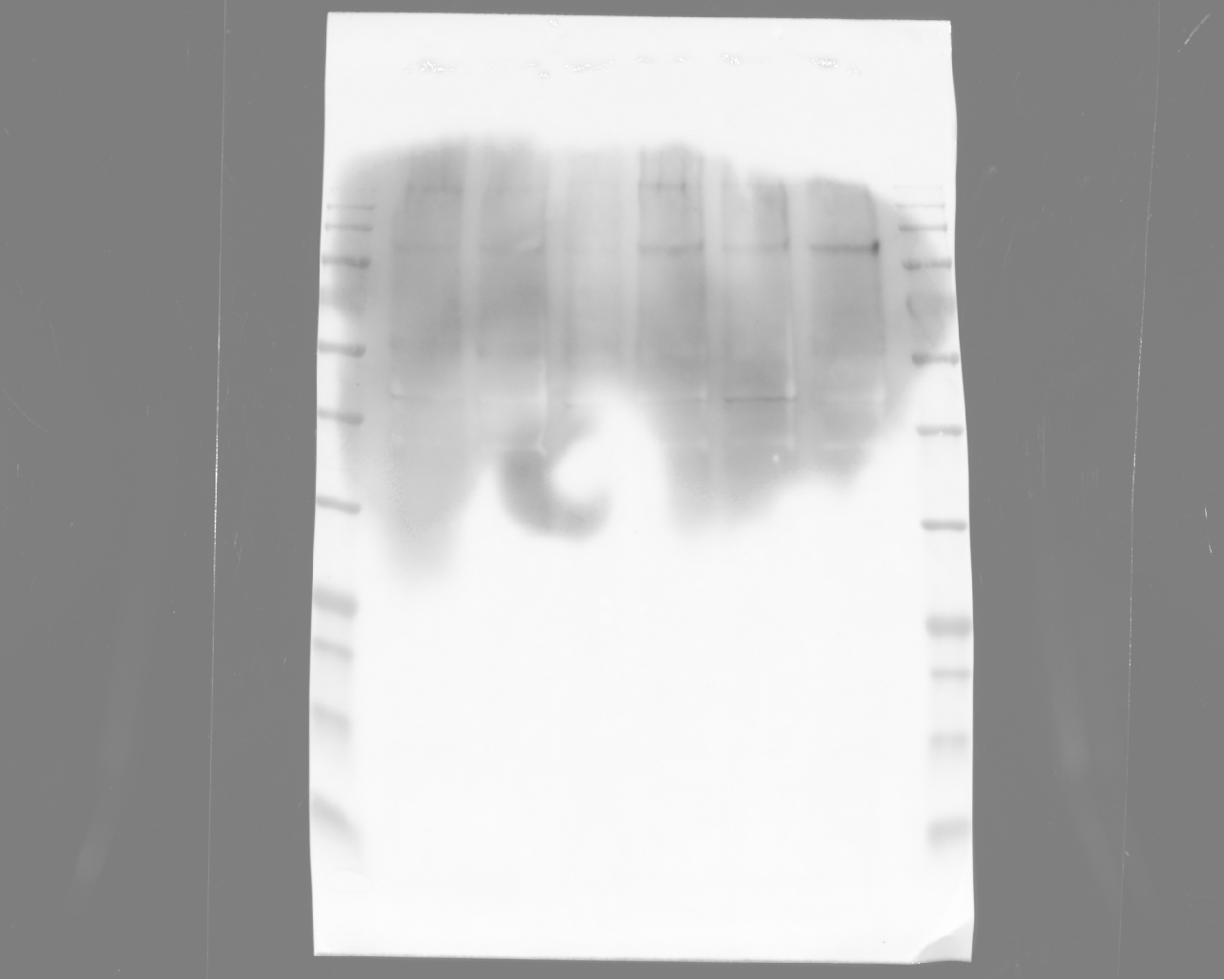

Supplement: Figure 2—source data 2. [file elife-96076-fig2-data2.zip › Thbs4_raw.tif]

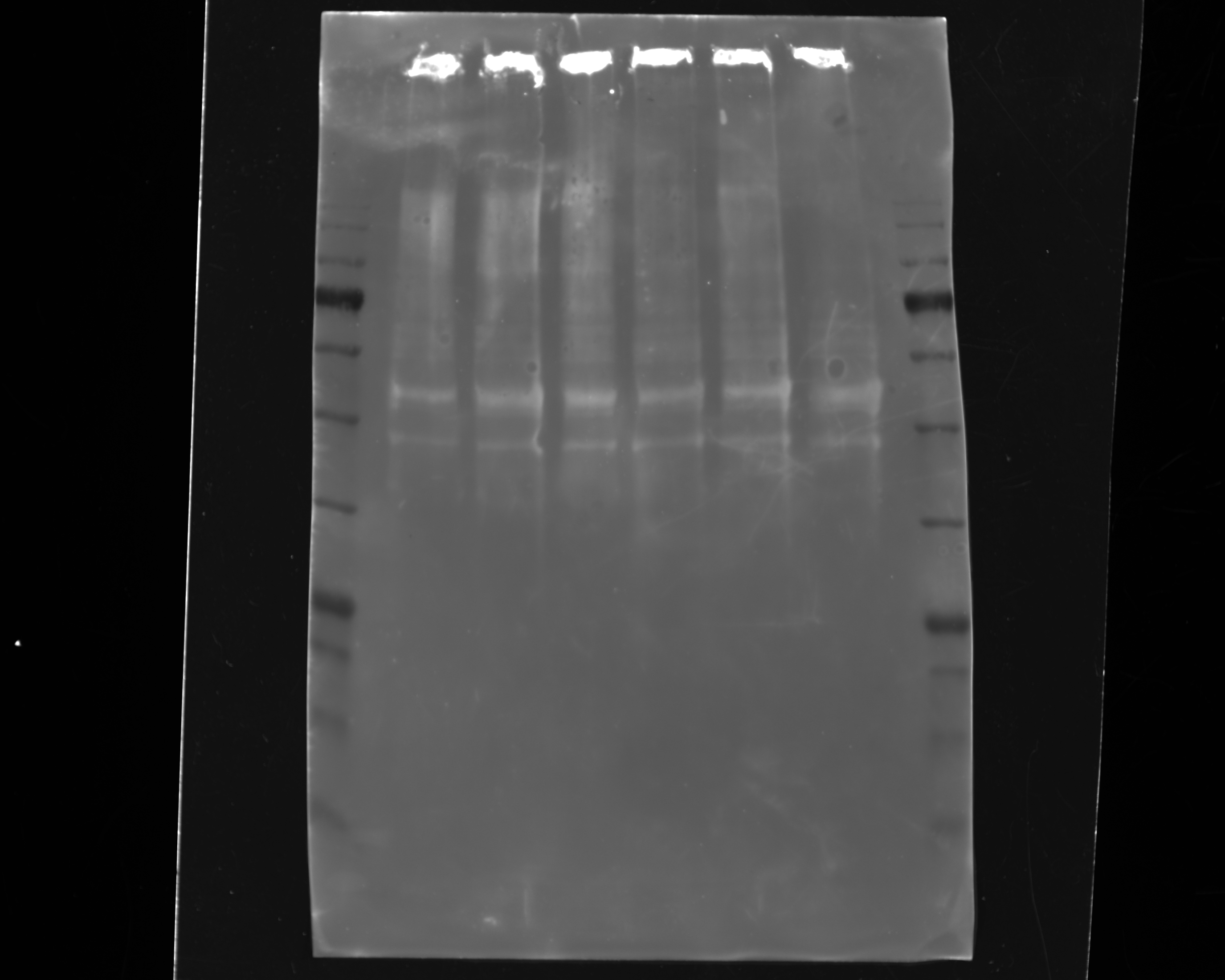

Supplement: Figure 2—source data 2. [file elife-96076-fig2-data2.zip › GAPDH_raw16.tif]

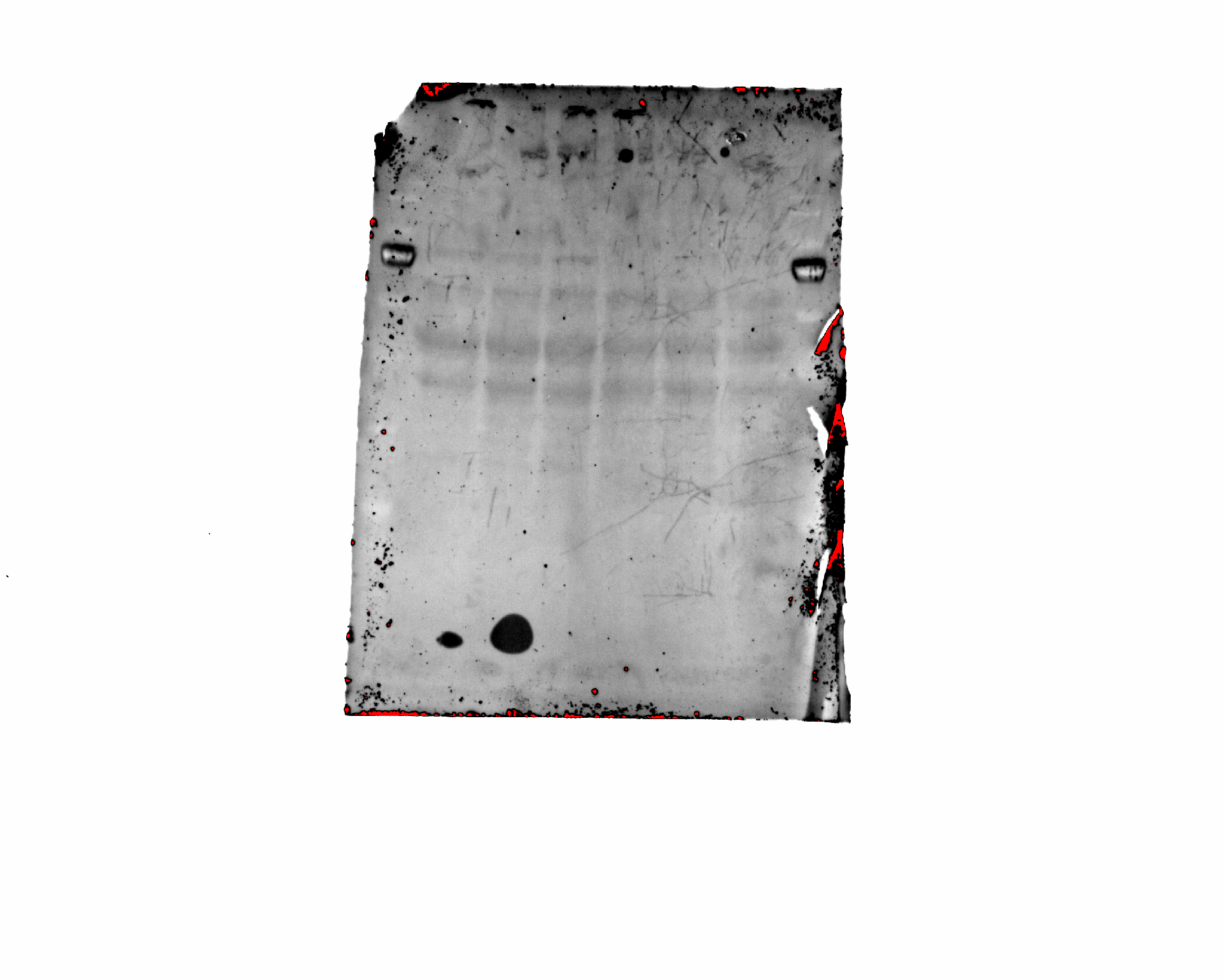

Supplement: Figure 2—source data 2. [file elife-96076-fig2-data2.zip › Nestin_raw.tif]
